# Public Awareness of the Emergency Medical Services in Maharashtra, India: A Questionnaire-based Survey

**DOI:** 10.7759/cureus.3309

**Published:** 2018-09-15

**Authors:** Pranav D Modi, Rajavi Solanki, Tripti S Nagdev, Pallavi D Yadav, Nyayosh K Bharucha, Ajay Desai, Paresh Navalkar, Sunil B Kelgane, Deepak Langade

**Affiliations:** 1 EMS Officer, Lifesupporters Institute of Health Sciences, Mumbai, IND; 2 Intern, D.Y. Patil Medical University, Mumbai, IND; 3 Anaesthesiology, D.Y. Patil Medical College and Hospital, Mumbai, IND; 4 EMS Officer, Lifesupporters Institute of Health Sciences, Navi Mumbai, IND; 5 EMS Officer, Maharashtra Emergency Medical Services, Pune, IND; 6 Pharmacology, D.Y. Patil University School of Medicine, Navi Mumbai, IND

**Keywords:** ems awareness, india ems, 108, 112

## Abstract

Background and objectives

The most widely used emergency medical services (EMS) model in India is the ‘108’ emergency service which primarily functions as an emergency response system to attend patients in need of critical care, trauma and accident victims. This is an observational cross-sectional study which was conducted using a questionnaire that asks the participants about their awareness and opinion of the current EMS system. The results of this study will enable us to ascertain the level of awareness of EMS among the population and address any misconceptions if they exist.

Materials and methods

All participants had to complete a 24-item self-administered questionnaire consisting of eight socio-demographic questions and 16 questions based on the EMS system. Questions regarding the development of the ‘112’ unified emergency service were also included. The convenient sampling method was used for data collection. The distribution of responses was examined using frequencies and percentages. Further analysis was done using the Chi-square test to compare responses between various subgroups based on the age, gender, profession, and level of education.

Results

A total of 1220 people from the state of Maharashtra responded to the survey and the maximum responses were from Mumbai. Majority of the respondents (59.2%) were from the age group of 15 to 30 years and, most of our responders had received education at the graduate level or above (78.2%). Only 17.5% of the respondents said that they will try to check for responsiveness if they saw a person lying unconscious by the side of the road with the scene being free of any danger. Interestingly, 78.9% of the healthcare professionals who participated in this survey would not check for responsiveness. Only 76.2% of the respondents knew that '108' is the number to dial in case of a medical emergency and about a quarter of them was not aware of it. It may seem that a good number of people are aware of the number. However, with the high number of fatalities occurring every day due to lack of medical facilities and a high current annual death toll on the roads, 100% of the population should know the emergency number. Only 20.2% of the respondents had called the EMS and asked for an ambulance. 68.5% of the respondents would immediately move out of the way and 27.5% of them would move out of their way if the ambulance’s lights and sirens were on. About two-thirds of the respondents were unaware of the development of a unified emergency number (112). However, a large majority (82.9%) were in favor of having a unified emergency number instead of a different number for each emergency. Only 43.8% of the respondents were of the opinion that the current EMS coverage was inadequate. 24.9% of the participants rated the current EMS as good, whereas 53.5% rated the EMS average and 16.9% rated it poor.

Conclusions

An effort should be made to make 100% of the population aware of this service. The first step for increasing awareness would be starting various advertisement campaigns. The next step would be to implement the unified emergency number (112) to address all kinds of distress calls such as police, fire, and ambulance. A very small proportion of the population is trained in first-aid or basic trauma life support. Awareness campaigns and training sessions for the general public should be conducted for the same. It is also necessary to spread awareness and help the populace know about the Good Samaritan law.

## Introduction

With a population of over 1.3 billion people, India is the second most populated country in the world. Healthcare has seen a major transformation in India in the last few years with the setup of numerous hospitals, ambulance services, and improved medical facilities, especially in the urban areas. However, the current emergency medical services (EMS) system in India is still in its nascent stages. Most of the states in India have initiated various models of EMS which have been supported under the National Health Mission (NHM). The most widely used model in India is the ‘108’ emergency service which primarily functions as an emergency response system to attend patients in need of critical care, trauma and accident victims. The ‘102’ ambulance service mainly deals with basic patient transport which also includes the free transfer to home facility, intercity transfer facility for referrals, drop back facility for mother and children [1]. The EMS is publicly financed across all states but the mode of delivery varies. The Maharashtra Emergency Medical Services (MEMS) is a project under National Rural Health Mission (NRHM) and implemented and operated by BVG India Ltd. across the state of Maharashtra [2].

With the ‘108’ and ‘102’ services in place, it is important to know if people are aware of the course of action to take when confronted with a medical emergency. Even though the EMS scenario is consistently improving in the urban areas, the lack of basic medical facilities and limited information about EMS among the rural population poses a major challenge in developing an organized EMS system in these regions which would cater to more than 70% of the Indian population.

Studies assessing the public awareness of EMS have been conducted in many parts of the world but very few have been reported from India. A study conducted among 1534 people in the Western region of the Kingdom of Saudi Arabia showed that 33% of the people were unaware of the number to call in case of a medical emergency [3]. Four hundred and sixty-eight people from Accra, Ghana were surveyed to assess their use of the public access medical emergency telephone number. Only 43.8% of the responders were aware of the number and 37.1% of them knew that it was toll-free [4]. One thousand two hundred participants from the general population of Japan were surveyed to assess their awareness of the need to call EMS following the onset of an acute myocardial infarction (MI). Only 11.6% of the participants stated that they would call EMS during on-time hours (daytime), and 27.5% stated that they would call during off-time hours (nights/holidays) [5]. In a cross-sectional study conducted among 1680 residents of Shiraz, 48.6% of them were not aware of EMS [6]. A survey was conducted by Vasudevan et al. to understand the effectiveness of ‘108’ services from a traffic safety perspective in India. The results showed that the population is unaware of the ‘108’ services and despite having access to it is not frequently utilized to transport victims of traffic crashes [7].

This study was a cross-sectional study which was conducted using a questionnaire that asked the participants about their awareness and opinion of the current EMS system. The results of this study will enable us to ascertain the level of awareness of EMS among the population and address any misconceptions if they exist. Questions regarding awareness of the recently developed ‘112’ unified emergency service number have also been included in the survey [8].

## Materials and methods

The study is a cross-sectional survey to assess the awareness of the current EMS system in India. It is important that the majority of the population is aware of the EMS system. Hence any individual residing within the boundaries of India was eligible to participate in the survey. Participants residing out of India were excluded from the survey.

All participants had to complete a 24-item self-administered questionnaire consisting of eight socio-demographic questions and 16 questions based on the EMS system. This questionnaire consisted of questions adapted from a study assessing the public awareness of the EMS system in Western Saudi Arabia and also other questions which were more relevant to the Indian population, addressing local and cultural issues [3]. Questions regarding the development of the ‘112’ unified emergency service were also included [8]. The convenient sampling method was used for data collection and an effort was made to interview a large number of people from all walks of life. Healthcare professionals, patients in the outpatient clinics, various non-medical professionals, students, homemakers, and retired individuals were interviewed. The questionnaire was to be filled by the participants in either print (paper) format or online. For the participants who could not be interviewed in person, an online link to the survey was available to them which was distributed on various social media platforms. Consent was obtained from all responders and their participation was voluntary. Completed questionnaires were coded and the data was tabulated prior to analysis. The distribution of the responses was examined using frequencies and percentages. Further analysis was done using the chi-square test to compare responses between various subgroups based on the age, gender, profession, and level of education.

## Results

The responses of participants to the survey on public awareness of EMS are provided in Tables 1-16.

**Table 1:** What would you do next if a person is lying unconscious by the side of the road with the scene being safe?

	Number of Respondents	Percent
Call the emergency response services	563	46.1
Call the people standing nearby	118	9.7
Call the police	46	3.8
Ignore and walk away	16	1.3
Shift to the nearby hospital	264	21.6
Try to awaken the person	213	17.5
Total	1220	100.0

**Table 2:** Are you aware of the toll-free number to call in case of a medical emergency?

	Number of Respondents	Percent
100	86	7.0
101	204	16.7
108	930	76.2
Total	1220	100

**Table 3:** Are you aware of the development of a unified number 112 to call in case of an emergency?

	Number of Respondents	Percent
No	806	66.1
Yes	414	33.9
Total	1220	100.0

**Table 4:** Do you think that having a unified number is better, or a different number for each service is better?

	Number of Respondents	Percent
Multiple	209	17.1
Unified	1011	82.9
Total	1220	100

**Table 5:** Have you ever called the EMS and asked for an ambulance? EMS: Emergency Medical Services

	Number of Respondents	Percent
No	973	79.8
Yes	247	20.2
Total	1220	100

**Table 6:** If you were to call the EMS from your home now, how long do you think it would take them to arrive? EMS: Emergency Medical Services

	Number of Respondents	Percent
1 hour	199	16.3
10 minutes	159	13.0
15 minutes	371	30.4
30 minutes	491	40.2
Total	1220	100.0

**Table 7:** Do you think it is part of the paramedic's duty to treat patients at the scene or just to transport them?

	Number of Respondents	Percent
Treat	262	21.5
Transport	75	6.1
Both	883	72.4
Total	1220	100.0

**Table 8:** Do you think that the current EMS coverage for your city is adequate? EMS: Emergency Medical Services

	Number of Respondents	Percent
No	808	66.2
Yes	412	33.8
Total	1220	100.0

**Table 9:** Do you think we need an air ambulance service at this stage?

	Number of Respondents	Percent
No	224	18.4
Yes	996	81.6
Total	1220	100.0

**Table 10:** Do you trust the EMS to handle your family members in a medical emergency? EMS: Emergency Medical Services

	Number of Respondents	Percent
No	306	25.1
Yes	914	74.9
Total	1220	100.0

**Table 11:** Which of the following vehicles would be appropriate to transport a dead person?

	Number of Respondents	Percent
Ambulance	574	47.0
Hearse	596	48.9
Private vehicle	50	4.1
Total	1220	100.0

**Table 12:** Do you think that a male paramedic should be allowed to respond to a female patient in the absence of a male family member?

	Number of Respondents	Percent
No	226	18.5
Yes	994	81.5
Total	1220	100.0

**Table 13:** Do you think that paramedics should have the right to refuse transport of stable patients?

	Number of Respondents	Percent
No	428	35.1
Yes	792	64.9
Total	1220	100.0

**Table 14:** Do you think medical insurance should cover the EMS/ambulance facility? EMS: Emergency Medical Services

	Number of Respondents	Percent
No	78	6.4
Yes	1142	93.6
Total	1220	100

**Table 15:** What would you do if you saw an ambulance coming up behind you on the road?

	Number of Respondents	Percent
I won't move, an ambulance should use the emergency lane and not the middle lane of the road	13	1.1
I would immediately get out of the way	836	68.5
Move only if the ambulance is behind me	36	3.0
Move out of the way only if lights and sirens are on	335	27.5
Total	1220	100.0

**Table 16:** What do you think of the current EMS service overall? EMS: Emergency Medical Services

	Number of Respondents	Percent
Average	653	53.5
Excellent	58	4.8
Good	304	24.9
Poor	205	16.8
Total	1220	100.0

A total of 1220 people responded to the survey. Any individual above the age of 15 years, residing within the boundaries of India, was included in the study analysis. Any individual who did not meet the criteria was excluded from the analysis. We made an attempt to reach out to a large number of people across India. However, most of the respondents were from the state of Maharashtra. Within Maharashtra, maximum responses were from Mumbai city. The recorded percentage of responders from South and Central Mumbai was 28.4%, 39.8% from Western Mumbai, 8.5% from Navi Mumbai, 16.6% from Thane district and 6.7% from rest of Maharashtra.

**Figure 1:**
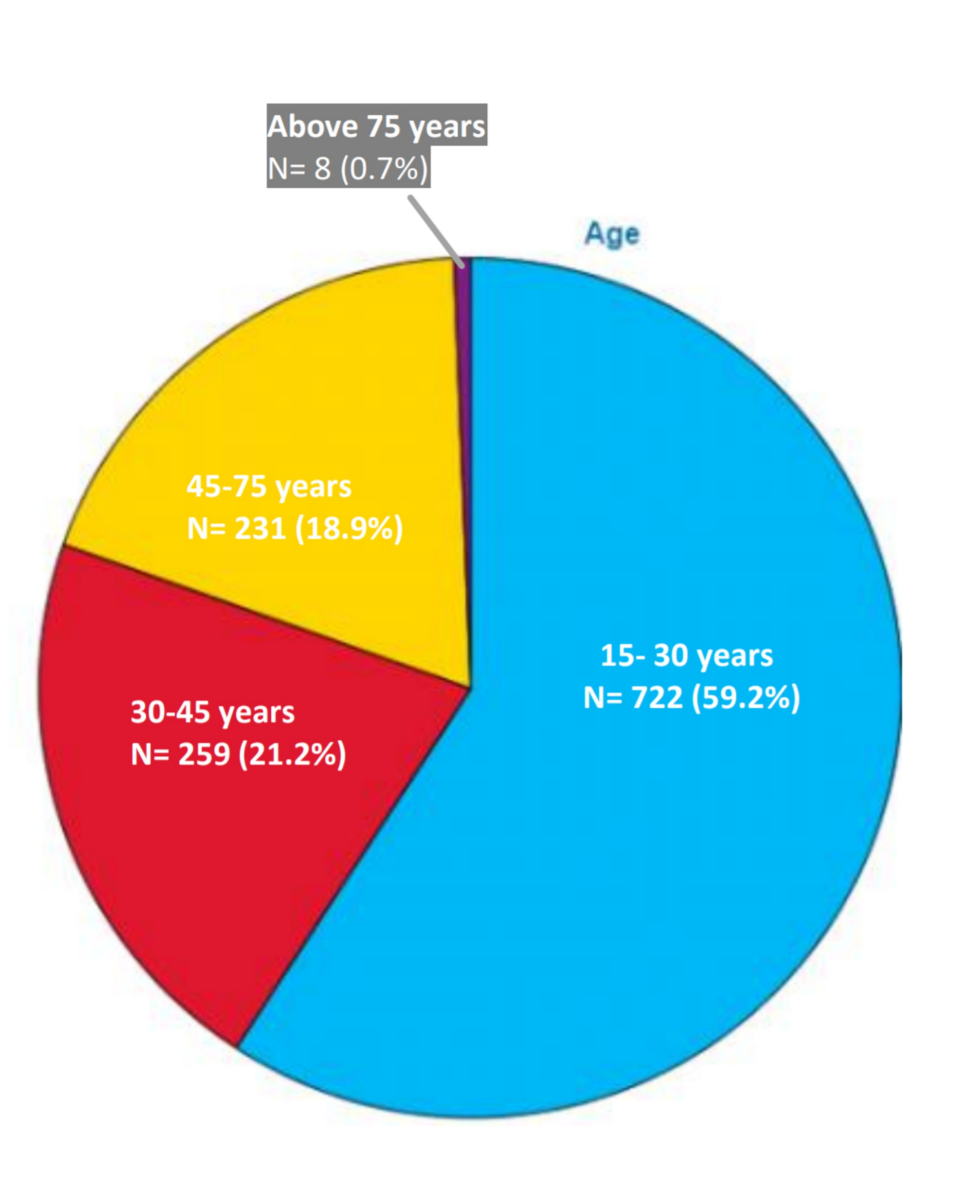
Distribution of responses according to age group.

**Figure 2:**
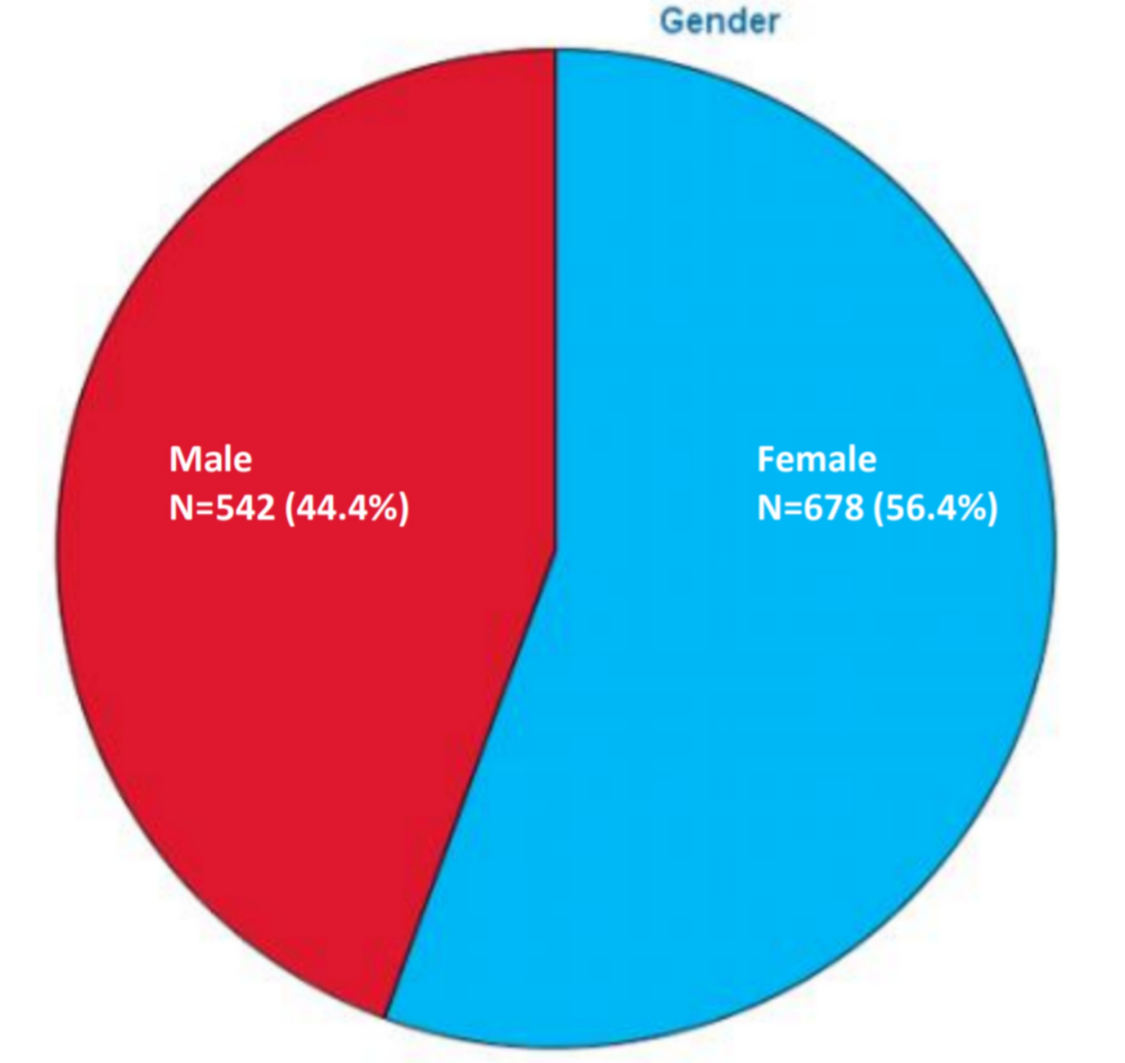
Distribution of responses according to gender.

**Figure 3:**
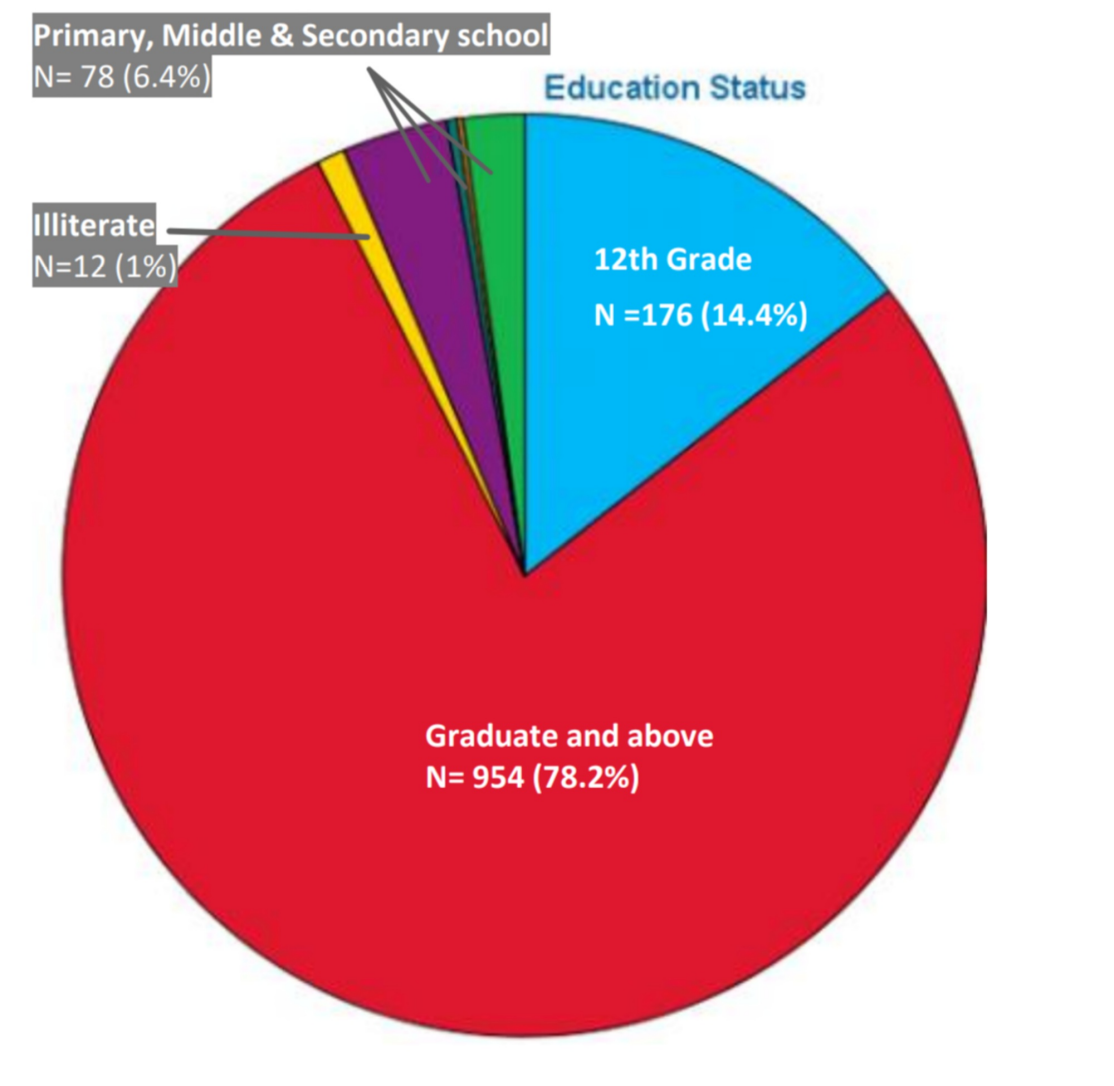
Distribution of responses according to the level of education.

**Figure 4:**
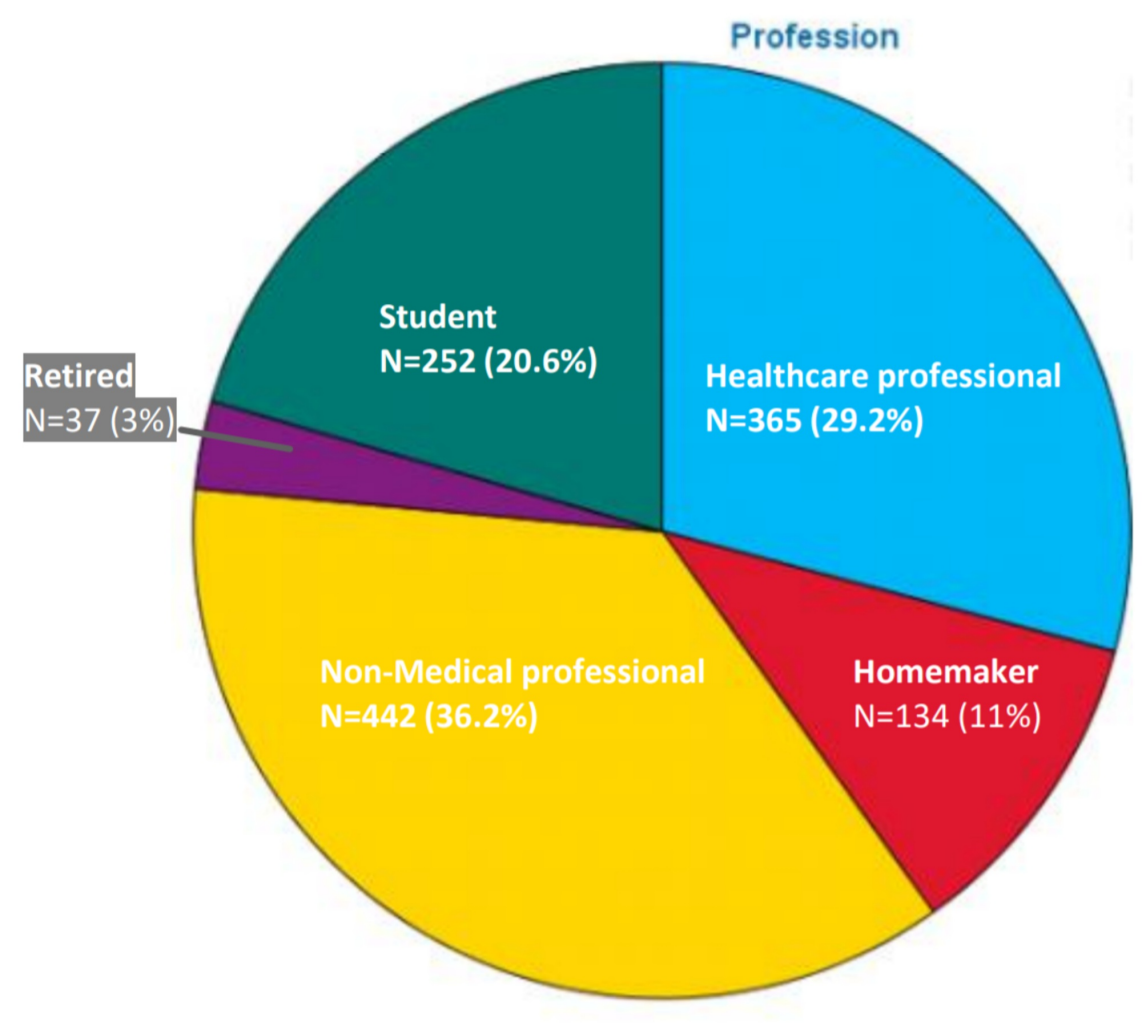
Distribution of responses according to the profession.

Majority of the responders (59.2%) were from the age group of 15 to 30 years, followed by the age groups of 30 to 45 years (21.2%), 45 to 75 years (18.9%) and above 75 years (0.7%) (Figure 1). A greater number of responses were from females (55.6%) as compared to males (44.4%) (Figure 2). As evident from Figure 3, most of our respondents had received education at the graduate level or above (78.2%), followed by 12th grade (14.4%) and primary, middle and secondary school (6.4%). A very small number of responders (n = 12) were illiterate who were interviewed personally. Figure 4 shows the distribution of participants according to the profession.

The remainder of the survey had questions based on the current EMS system. To start off, people were asked how they would react if they saw a person lying unconscious by the side of the road with the scene being free of any danger. Only 17.5% said that they will try to awaken the person and check for responsiveness. However, 46.1% said that they would call the EMS and 9.7% said that they would call people standing nearby. Only 21.6% people said that they would shift the person to a nearby hospital and a handful also said that they would ignore the situation and walk away. When responses from different age groups were compared, the maximum number of responses were from the age group of 15–30 years (n = 722). Only 21.1% of the responders from this group answered correctly as compared to 12.5% from the age group of 20–45 years, and 12.1% from the age group of 45–75 years (p = 0.01). Only eight responses were received from the age group of 75 and above and 25% of them answered correctly. More number of females answered correctly (20.4 %; n = 138) as compared to males (13.8%; n = 75) (p = 0.03). Interestingly 78.9% (n = 281) of the healthcare professionals and 84% (n = 726) of all other non-healthcare related professionals, students, homemakers, and retired people would not check for responsiveness. Respondents who had an education level of graduate and above gave the maximum correct responses (18.9%; n = 180), whereas none of the people who were illiterate could answer this question correctly. However, the total number of respondents belonging to this group was only 12. No significant difference was observed between responses from various groups based on the level of education. It was evident from the responses to this question that there is a lack of awareness regarding the steps of basic life support (BLS).

The following question was probably among the most important questions of this survey, asking the responders if they aware about the toll-free number to call in case of an emergency. It was observed that 76.2% correctly responded that ‘108’ is the number to call in case of a medical emergency. However, 16.7% said that the number was 101 and 7% thought that ‘100’ was the toll-free number for medical emergencies. Maximum number of correct responses were from the group of healthcare professionals 86.2% (n = 306) followed by 74.1% (n = 186) from students, followed by 71.6% (n = 96) from homemakers, 71% (n = 314) from non-medical/healthcare professionals (p < 0.0001). Seventy-three percent (n = 27) of correct responses were obtained from people who had retired, however, the total number of responses were only 37. Only 79.5% (n = 431) of the correct responses were from males whereas 73.6% (n = 499) were from females. Highest awareness regarding the toll-free number (75.1%; n = 542) was observed in the respondents from the age group of 30–45 whereas as the lowest awareness was seen in the age group of 75 and above (75%; n = 6). However, the total number of respondents from this age group was only eight. With regards to the level of education, the highest number of correct responses (77.8%; n = 742) were from respondents with an education level of graduate and above. The least number of correct responses were from people who were illiterate (58.3; n = 7). However, only 12 responses were obtained from this group. The differences between various subgroups based on age, gender, level of education for the awareness of the toll-free number were not significant. It was also interesting to note that the majority of the correct responses, 89% (n = 73), were from rest of Maharashtra but the total number of responses from this region was only 82. The number of correct responses from Rest of South and Central Mumbai was 263 (75.8%), 356 from Western suburbs of Mumbai (21.6%; n = 356), 87 (83.7%) from Navi Mumbai and 151 (74.8%) from Thane district. Interestingly, 79.8% (n = 973) of the respondents had never called the EMS and asked for an ambulance.

The survey also asked the respondents if they were aware of the development of a unified emergency number (112). About two-thirds of the responders were unaware of the development of ‘112’. However, a large majority (82.9%) were in favor of having a unified emergency number instead of a different number for each emergency.

It is also important to know whether people are aware of the roles and responsibilities of a paramedic and they were asked if the paramedic’s duty was to treat the patient, transport the patient to the nearest hospital or to do both. More than 72% of the responders believe that the paramedic’s duty is to treat and transport the patient to the nearest facility. When asked if a male paramedic should be allowed to respond to a female patient in case of a medical emergency in the absence of a male family member, 81.5% of the responders were in agreement, whereas 18.5% of them were against it. 64.9% of the respondents agree that paramedics should have the right to refuse transport patients who are stable and do not need emergency care. On the other hand, 35.1% of the responders were in disagreement. Majority of the people (74.9%) said that they trust the EMS to handle their family in a medical emergency, while about a quarter of them do not trust the EMS.

Traffic snarls are very common in India, especially in the metros. It can be very challenging at times for the EMS to navigate through the congested roads, especially when there is no provision of a dedicated emergency lane. The respondents were asked about what they would do if they saw an ambulance coming up behind them on the road. Only 68.5% of them said that they would immediately get out of the way, whereas 27.5% of them said that they would move out of their way if the ambulance’s lights and sirens were on. Three percent of them said that they would move only if the ambulance was behind them. A small number of responders (1.1%) also said that they would not move because they believe that ambulances should use an emergency lane and not the middle lane of the road. They were also asked about their thoughts regarding the time in which the EMS team would arrive at the scene of an emergency. Their responses were varied with 30.4% of them saying it would take 15 minutes and 40.2% of them saying it would take half an hour for the EMS team to arrive. There were a large number of responses in favor of starting an air ambulance at this stage.

Regarding the choice of vehicle to transport a dead person, only 48.9% of the people were aware that the hearse is the appropriate vehicle. Forty-seven percent said that an ambulance should be used whereas a small number of people also believed that a private vehicle would be appropriate to transport a dead person.

Questions asking the people about their opinion of the current EMS system were also included. Interestingly, 66.2% said that the current EMS system for their city is inadequate. Only 24.9% of the participants said the current EMS is good, whereas 53.5% rated the EMS as average and 16.9% rated it poor. More than 93% of the participants were in favor of the EMS/ambulance service being covered under medical insurance.

## Discussion

The objective of this survey was to ascertain the level of awareness regarding the current EMS system and to also find out what the general public knows and what they do not know regarding the current EMS system. It is evident from the results that only 76.2% of the respondents know that ‘108’ is the number to dial in case of a medical emergency and about a quarter of them was not aware of it. It may seem that a good number of people are aware of the number. However, with the number of fatalities occurring every day due to lack of medical services and the current annual death toll on the roads of over 140,000, 100% of the population should know the emergency numbers. Also, only three-fourths of the participants trust the EMS to handle their family members. It was surprising to note that only 20.2% of the respondents had called the EMS and asked for an ambulance whereas the rest had never used this service. The possible reasons for this could be:

·         Lack of awareness among the general population regarding the ‘108’ service and its scope

·         Medical conditions that are covered under this service

·         Not knowing how the EMS system actually works

·         Lack of knowledge about the steps of basic life support (BLS) and activating the EMS

·         Fear of being subjected to legal hassles, repeated police questioning and multiple court appearances

·         Did not face a situation that needed them to activate the EMS

Let’s take a moment to discuss the points mentioned above. What is the scope of the ‘108’ service and how does it function? Under the Government of Maharashtra and the National Rural Health Mission (NRHM), the Maharashtra Medical Emergency Service (MEMS) project provides pre-hospital care via life support ambulances in collaboration with BVG India Limited. This service includes the following conditions:

·         Road and all other accidents, assault, burns

·         Natural calamities and man-made hazards

·         All critical diseases

·         Mass casualties

·         Labor/pregnancy

·         Childbirth/neonatal disorders

·         Patients affected by epidemics

·         Patients with critical cardiovascular disease

·         Snake bites, poisoning, intoxication

·         Brain and respiratory diseases

·         Other disorders

It is observed from the detailed report on MEMS type of emergencies served in 2014–2016 that after medical emergencies the most common emergency served was related to labor/pregnancy, followed by accident cases and intoxication and poisoning. With a good number of medical emergencies covered by this service, it is important to make the general population aware of the same [9].

Well trained paramedics, doctors, nurses and other staff on board are essential for the smooth functioning of the '108' service. For the ‘108’ service in Maharashtra, the Emergency Response Center (ERC) located in the city of Pune provides training for doctors, ambulance drivers, call takers and call dispatchers for operationalization of EMS. Doctors are mainly trained for BLS, advanced cardiac life support (ACLS), and disaster management. As per the data available on the National Health Mission Maharashtra website, a total of 937 ambulances have been allocated for the state of Maharashtra out of which 704 are BLS ambulances and 233 are ACLS ambulances. Also, 690 ambulances are dedicated for the rural areas in Maharashtra [9].

The strategy for the operation of this service is based on the “Golden Hour Theory”, where the patient must be shifted within the first hour to the nearest hospital. The system works with an integrated approach that includes computer technology integration, voice logger system, Mobile Communication System (MCS), GIS (Geographic Information System), GPS (Global Positioning System), AVLT (Automatic Vehicle Location System). Calls dialed to ‘108’ from across Maharashtra reach the ERC, from where trained call handlers understand the emergency and dispatch the nearest ambulance to rescue the patient. Medical equipment in the ambulance includes an ambulance cot, scoop stretcher, bi-phasic defibrillator cum cardiac monitor with a recorder and transport ventilator (for advanced life support (ALS) ambulances only), pulse oximeter (for BLS ambulances only), suction pump, and an oxygen cylinder [9].

Another important factor in activating the EMS is knowing the steps of BLS. In this survey, it was observed that only 17.5% of the respondents would try to awaken and check for responsiveness in a person lying unconscious by the side of the road, after verifying scene safety. It was surprising to note that 78.9% of the respondents who were healthcare professionals would not check for responsiveness in an unconscious patient. This points towards the need of spreading awareness regarding Basic Life Support skills among the general population as well as healthcare professionals. According to the BLS Healthcare Provider Adult Cardiac Arrest Algorithm—2015 update, the first step to approach an unconscious patient is to verify scene safety. Next would be to check for responsiveness. If the victim is not responsive, shout for help and activate the EMS [10]. 46.1% of the respondents in our survey would call the EMS and 9.7% said would call people standing nearby. This question was adapted from a study evaluating the awareness among general population towards common medical emergencies and basic life support skills among 445 participants randomly selected from visitors of the outpatient department of Internal Medicine, SKIMS Medical College in Srinagar, India. Similar to the results of our study, only 16% of the respondents would check for responsiveness in an unconscious patient. The results of this study showed awareness about common medical emergencies is low in the general public and there is a need to devise strategies to improve this awareness [11]. Besides BLS training in adults, many studies have supported the need for early awareness and training of Basic Life Support and resuscitation techniques in school children [12-14]. When the responses to this question were analyzed according to different age groups, it was observed that individuals with an education level of graduate and above had the most number of correct responses. Twelve individuals in this study were illiterate and none of them could answer this question correctly. However, since responses were obtained only from 12 individuals, there is a need to interview more people from this group.

Only 1.3% of the respondents said that they would ignore the victim and walk away. It was also discussed earlier that 79.8% of the participants had never called the EMS and asked for an ambulance. The possible reason for this trend of responses could be fear among the public of being subjected to legal hassles, repeated police questioning, and multiple court appearances. A study on impediments to bystander care in India was conducted by SaveLIFE foundation in 2013 in major cities across India. The results of this study showed 74% of the bystanders are unlikely to assist a victim of serious injury irrespective of whether they are alone on the spot. Eighty-eight percent of these respondents stated that they were reluctant to help for fear of legal hassles, including repeated police questioning and court appearances. Eighty-eight percent of the respondents also expressed the need for a supportive legal environment to enable Good Samaritans to come forward and help injured victims on the road [15]. On March 30, 2016, the Supreme Court of India gave “force of law” to the guidelines for the protection of Good Samaritans issued by the Ministry of Road Transport and Highways. The purpose of this law is to provide legal protection to bystanders who come to the aid and rescue of victims of road crashes. It is of utmost importance to spread awareness and help the public know about this law. According to the WHO, over 70,000 lives can be potentially saved if bystanders come forward to help [16].

In today’s date, traditions and customs still have an important place in India. 81.5% of the respondents were in agreement when asked if a male paramedic should attend a female victim in the absence of a male family member. However, 18.5% of them were against it. These results are similar to results of the study assessing public awareness of EMS in western Saudi Arabia. Only 17.7% of the respondents in this study did not find it acceptable for a male paramedic to attend a female victim in case of medical emergency in absence of a male family member [3]. These results may not seem relevant especially in the western world. However, they are important in regions where culture and tradition override the need to provide emergency medical treatment to a victim.

**Figure 5:**
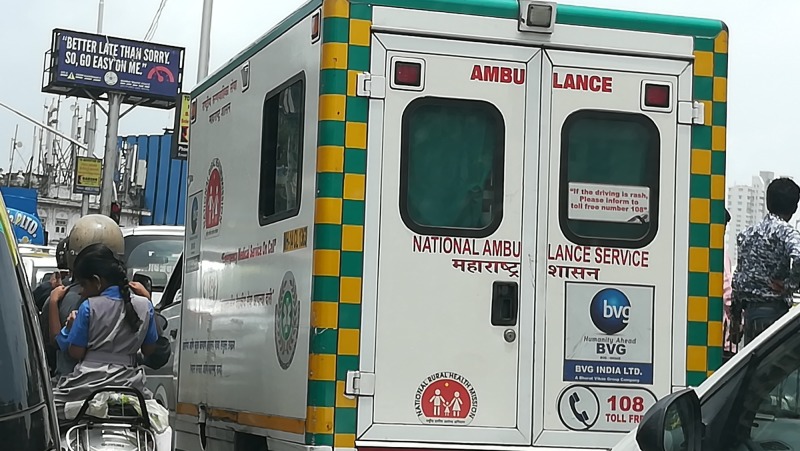
The ‘108’ ambulance navigating through traffic in Mumbai.

With regards to the transport of a victim to the nearest hospital, traffic in India, especially in the metros can be a major hurdle in providing effective EMS (Figure 5). Besides a large number of vehicles on the road, traffic rules are not diligently followed by motorists in India. With this being said, it was interesting to note that 68.5% of the respondents would immediately move out of the way and 27.5% of them would move out of their way if the ambulance’s lights and sirens were on. The MEMS in collaboration with BVG recently came up with an innovative idea to deal with the issue of traffic. “Doctors on bikes” are now available in certain parts of the state who have been trained to handle road traffic accidents, cardiac arrest cases, deliveries, and respiratory distress as four major specialties under this service. The expected time for the '108' ambulance to reach the scene is 20 minutes in the city and 30 minutes in the rural setting. In case of the doctor on bike service, the doctor is dispatched on a motorbike simultaneously with an ambulance that follows as soon as a call is received from the scene of the incident. The motorbike is equipped with a portable stretcher, delivery kit, emergency trauma kit and basic airway management kit with oxygen support. The doctor on the bike can reach the scene and stabilize the patient by the time the ambulance can reach to transport the victim to the nearest hospital. This further cuts down the time in the golden hour [17]. Another major development that is required is a dedicated emergency lane to ensure the patient is shifted within the golden hour to the nearest hospital, thereby reducing mortality.

Only 49% of the participants were aware that the hearse is the appropriate vehicle to transport a dead person. A recent report suggested a shortage of hearses in Mumbai has allowed private ambulance operators to transport the dead at a hiked up price. It was also observed that the people were not informed by staff at hospitals and morgues regarding the hearse service provided by the local municipal corporation for a nominal fee. An effort to increase awareness among the public regarding this service especially at morgues and hospitals is needed [18].

Respondents were asked regarding their overall opinion of the current EMS. Only 43.8% were of the opinion that the current EMS coverage was inadequate for their city whereas 62.8% felt the services are adequate. However, only 24.9% of the participants said the current EMS is good, whereas 53.5% rated the EMS as average and 16.9% rated it poor.

## Conclusions

An effort should be made to make 100% of the population aware of this service by starting various advertisement campaigns, preferably in the local language. The next step would be to implement the unified emergency number ‘112’, which is being referred to as India’s version of ‘911’. It was also evident from our study that respondents lacked awareness regarding the steps of BLS and provisions should be made to educate the general population and healthcare professionals regarding first aid, cardiopulmonary resuscitation (CPR) and BLS. It is also necessary to spread awareness and help the public know about the Good Samaritan law which would help alleviate the fear of legal repercussions and procedural hassles among bystanders. One of the drawbacks of this study is that the data is skewed to more respondents from bigger cities like Mumbai, Navi Mumbai, and its suburban areas which do not truly represent the entire state. Majority of the participants had an education level of graduate and above and more participants who have a lower level of education should also be interviewed.
